# (*S*)-*N*-[(4-{(*S*)-1-[2-(4-Meth­oxy­benz­amido)-2-methyl­propano­yl]pyrrolidine-2-carboxamido}-3,4,5,6-tetra­hydro-2*H*-pyran-4-yl)carbon­yl]proline dimethyl sulfoxide monosolvate (4-MeBz-Aib-Pro-Thp-Pro-OH)

**DOI:** 10.1107/S1600536813004546

**Published:** 2013-02-20

**Authors:** Svetlana A. Stoykova, Anthony Linden, Heinz Heimgartner

**Affiliations:** a Institute of Organic Chemistry, University of Zürich, Winterthurerstrasse 190, CH-8057 Zürich, Switzerland

## Abstract

The asymmetric unit of the title compound, C_28_H_38_N_4_O_8_·C_2_H_6_OS, contains one tetra­peptide and one disordered dimethyl sulfoxide (DMSO) mol­ecule. The central five-membered ring (Pro^2^) of the peptide mol­ecule has a disordered envelope conformation [occupancy ratio 0.879 (2):0.121 (2)] with the envelope flap atom, the central C atom of the three ring methylene groups, lying on alternate sides of the mean ring plane. The terminal five-membered ring (Pro^4^) also adopts an envelope conformation with the C atom of the methylene group closest to the carboxylic acid function as the envelope flap, and the six-membered tetra­hydro­pyrane ring shows a chair conformation. The tetra­peptide exists in a helical conformation, stabilized by an intra­molecular hydrogen bond between the amide N—H group of the heterocyclic α-amino acid Thp and the amide O atom of the 4-meth­oxy­benzoyl group. This inter­action has a graph set motif of *S*(10) and serves to maintain a fairly rigid β-turn structure. In the crystal, the terminal hy­droxy group forms a hydrogen bond with the amide O atom of Thp of a neighbouring mol­ecule, and the amide N—H group at the opposite end of the mol­ecule forms a hydrogen bond with the amide O atom of Thp of another neighbouring mol­ecule. The combination of both inter­molecular inter­actions links the mol­ecules into an extended three-dimensional framework.

## Related literature

For the azirine/oxazolone method, see: Heimgartner (1991 ▶); Altherr *et al.* (2007 ▶); Stamm & Heimgartner (2004 ▶). For the synthesis of Thp-containing peptides *via* the azirine/oxazolone method and their crystal structures, see: Suter *et al.* (2000 ▶). For the synthesis of Aib-Pro containing peptides *via* azirine coupling, see: Luykx *et al.* (2003 ▶); Stamm & Heimgartner (2006 ▶); Pradeille *et al.* (2012 ▶); Stoykova *et al.* (2012 ▶). For the insertion of Xaa-Pro units (Xaa = heterocyclic α-amino carb­oxy­lic acid) into peptides, see: Suter *et al.* (2000 ▶); Stamm *et al.* (2003 ▶). For the conformation of peptides containing α,α-disubstituted α-amino acids, see: Prasad & Balaram (1984 ▶); Toniolo & Benedetti (1991 ▶); Schweitzer-Stenner *et al.* (2007 ▶); Aravinda *et al.* (2008 ▶); Demizu *et al.* (2012 ▶). For crystal structures of peptaibols, see: Whitmore & Wallace (2004 ▶), authors of The Peptaibol Database http://www.cryst.bbk.ac.uk/peptaibol. For graph-set theory, see: Bernstein *et al.* (1995 ▶).
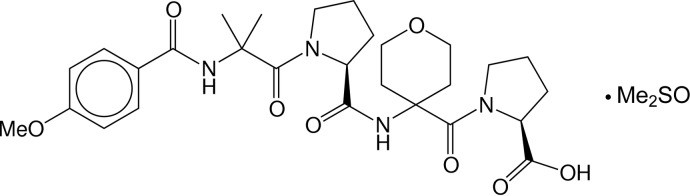


## Experimental

### 

#### Crystal data

C_28_H_38_N_4_O_8_·C_2_H_6_OS
*M* *_r_* = 636.76
Orthorhombic, 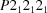

*a* = 10.8594 (1) Å
*b* = 13.7414 (2) Å
*c* = 21.1929 (3) Å
*V* = 3162.48 (7) Å^3^
*Z* = 4
Mo *K*α radiation
μ = 0.16 mm^−1^
*T* = 160 K
0.28 × 0.20 × 0.18 mm

#### Data collection

Nonius KappaCCD area-detector diffractometer
53400 measured reflections
9238 independent reflections
7711 reflections with *I* > 2σ(*I*)
*R*_int_ = 0.044

#### Refinement

*R*[*F* ^2^ > 2σ(*F* ^2^)] = 0.042
*wR*(*F* ^2^) = 0.103
*S* = 1.02
9231 reflections
433 parameters
21 restraints
H atoms treated by a mixture of independent and constrained refinement
Δρ_max_ = 0.28 e Å^−3^
Δρ_min_ = −0.33 e Å^−3^
Absolute structure: Flack & Bernardinelli (1999 ▶, 2000 ▶), 4115 Friedel pairs
Flack parameter: −0.02 (8)

### 

Data collection: *COLLECT* (Nonius, 2000 ▶); cell refinement: *DENZO-SMN* (Otwinowski & Minor, 1997 ▶); data reduction: *DENZO-SMN* and *SCALEPACK* (Otwinowski & Minor, 1997 ▶); program(s) used to solve structure: *SHELXS97* (Sheldrick, 2008 ▶); program(s) used to refine structure: *SHELXL97* (Sheldrick, 2008 ▶); molecular graphics: *ORTEPII* (Johnson, 1976 ▶); software used to prepare material for publication: *SHELXL97* and *PLATON* (Spek, 2009 ▶).

## Supplementary Material

Crystal structure: contains datablock(s) I, global. DOI: 10.1107/S1600536813004546/nc2305sup1.cif

Structure factors: contains datablock(s) I. DOI: 10.1107/S1600536813004546/nc2305Isup2.hkl

Supplementary material file. DOI: 10.1107/S1600536813004546/nc2305Isup3.cdx

Supplementary material file. DOI: 10.1107/S1600536813004546/nc2305Isup4.cml

Additional supplementary materials:  crystallographic information; 3D view; checkCIF report

## Figures and Tables

**Table 1:** Hydrogen-bond geometry (Å, °)

*D*—H⋯*A*	*D*—H	H⋯*A*	*D*⋯*A*	*D*—H⋯*A*
O2—H2⋯O7^i^	0.87 (2)	1.81 (3)	2.6669 (17)	168 (2)
N6—H6⋯O13	0.87 (2)	2.19 (2)	3.0468 (17)	169.5 (18)
N12—H12⋯O4^ii^	0.80 (2)	2.37 (2)	3.1247 (18)	156 (2)

Symmetry codes: (i) 
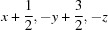
; (ii) 
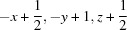
.

**Fig. 1.**
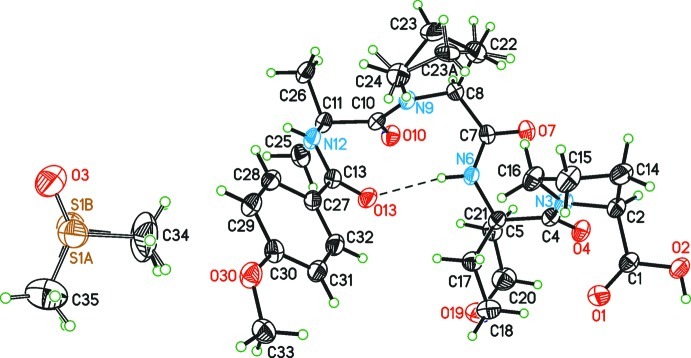
The molecular structure of the title compound showing the atom-labelling scheme and the intramolecular hydrogen bond (dashed line). Displacement ellipsoids are drawn at the 50% probability level. H atoms are represented by circles of arbitrary size and the alternate conformations of the disordered proline ring (atom C23) and DMSO molecule are shown by full and open bonds.

**Fig. 2.**
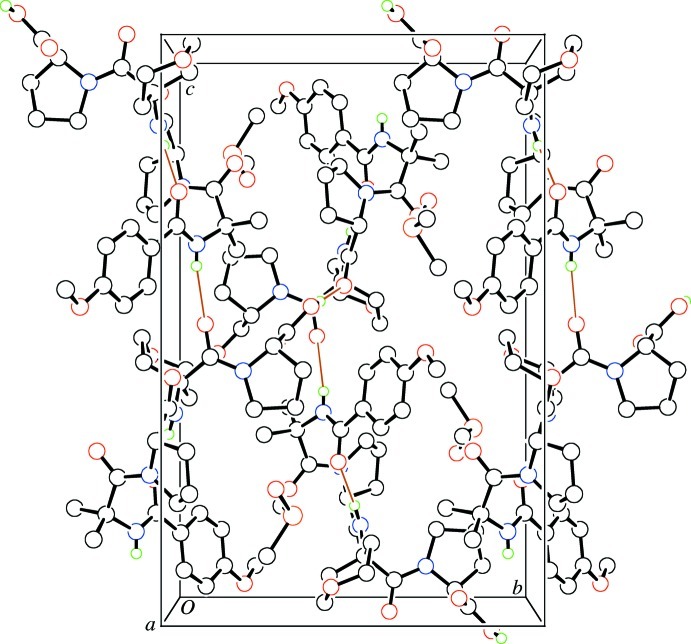
The crystal packing in the title compound viewed down the a axis and showing the N—H···O and O—H···O hydrogen bonds as thin brown lines.

## supplementary crystallographic information

### Comment

Peptaibols are naturally occurring peptides containing high proportions of α-aminoisobutyric acid (Aib) and occasionally other 2,2-disubstituted glycines (Whitmore & Wallace, 2004). As a result of the presence of α,α-disubstituted α-amino acids, these peptides adopt fairly rigid helical structures, the preferred conformation being the 3_10_-helix as a sequence of β-turns (Prasad & Balaram, 1984; Toniolo & Benedetti, 1991; Schweitzer-Stenner *et al.*, 2007; Aravinda *et al.*, 2008; Demizu *et al.*, 2012). In the past we have elaborated the 'azirine/oxazolone method' as a convenient protocol for the introduction of α,α-disubstituted α-amino acids into peptide chains in solution (Heimgartner, 1991; Altherr *et al.*, 2007) as well as on solid phase (Stamm & Heimgartner, 2004). In addition, it has been shown that the dipeptide unit Aib-Pro, which frequently appears in natural peptaibols (Whitmore & Wallace, 2004), can be inserted conveniently into a peptide chain *via* 'azirine coupling' with methyl *N*-(2,2-dimethyl-2*H*-azirin-3-yl)prolinate (Luykx *et al.*, 2003; Pradeille *et al.*, 2012; Stoykova *et al.*, 2012). In a similar manner, dipeptide segments consisting of a heterocyclic α-amino carboxylic acid and proline have been inserted into peptides *via* 'azirine coupling' (Suter *et al.*, 2000; Stamm *et al.*, 2003). In all cases, the heterocyclic α-amino carboxylic acid behaves in a similar way to other α,α-disubstituted α-amino acids, that is they induce helical conformations of the peptide. The synthesis of the title tetrapeptide was carried out with the aim of further testing the scope of the 'azirine coupling' with a combination of two different Xaa-Pro synthons.

The crystals of the title compound are enantiomerically pure and the expected absolute configuration, S at C2 and C8 of the two proline residues, has been confirmed by the diffraction experiment. The asymmetric unit contains one molecule of the peptide plus one molecule of DMSO. The S atom of the DMSO molecule is disordered over two sites (details in the Experimental section). The peptide molecule exists in a β-turn conformation stabilized by an intramolecular hydrogen bond between N6—H of the heterocyclic amino acid Thp and the O atom of the amide C=O group of the 4-methoxybenzoyl group (Fig. 1; Table 1). This interaction has a graph set motif (Bernstein *et al.*, 1995) of S(10). The central five-membered ring of Pro2 is disordered in that the ring has an envelope conformation in which atom C23 as the envelope flap is located on alternate sides of the mean ring plane with the major conformation found in 72.0 (10)% of the molecules. The other 5-membered ring (Pro4) also has an envelope conformation with atom C14 as the envelope flap. The six-membered tetrahydropyrane ring (Thp) exists in a chair conformation. All four amide groups are quite planar.

Classical intermolecular hydrogen bonds of the O—H···O and N—H···O type link the molecules into a three-dimensional framework (Fig. 2). This network is built from two substructures. In the first substructure, the carboxylic acid group, O2—H, forms an intermolecular hydrogen bond with one of the central amide O atoms, O7, of a neighbouring molecule, thereby linking the peptide molecules into extended chains which run parallel to the [100] direction and have a graph set motif of C(10). In the second substructure, the amide N—H group, N12—H, at the opposite end of the molecule forms an intermolecular hydrogen bond with the first amide O atom, O4, in the backbone of a different neighbouring molecule. This interaction links the peptide molecules into extended chains which run parallel to the [001] direction and have a graph set motif of C(11).

### Experimental

The title compound was prepared in analogy to earlier described procedures (Suter *et al.*, 2000; Stoykova *et al.*, 2012) by treatment of (*S*)-*N*-[*N*-(4-methoxyphenyl)-2-methylalanyl]proline (4-MeOBz-Aib-Pro-OH; Stoykova *et al.*, 2012) with two mol-equivalents of methyl (*S*)-*N*-(1-aza-6-oxaspiro[2.5]oct-1-en-2-yl)prolinate (Suter *et al.*, 2000) in dry THF at room temperature for 48 h. After removing the solvent under reduced pressure, the residue was purified by column chromatography (silica gel, CH_2_Cl_2_/MeOH; gradient 110:1 to 20:1). Saponification of the resulting tetrapeptide ester was achieved by treatment with 4 mol-equivalents of LiOH.H_2_O in THF/MeOH/H_2_O 3:1:1 at room temperature for 25 h. After completion of the reaction, 1*M* HCl was added until pH 1 was reached, and the organic solvent was evaporated. The residue was extracted with CH_2_Cl_2_, the combined organic phase was dried over MgSO_4_, and the solvent evaporated to give the title compound in 74% yield (over two steps). Colourless crystals suitable for an X-ray crystal structure analysis were grown from DMSO at *ca* 278 K.

### Refinement

The structure contains one molecule of DMSO per peptide molecule. The S atom of the DMSO molecule is disordered over two sites with the major orientation having a site occupation factor of 0.879 (2). One C atom of the central five-membered ring of the peptide molecule is also disordered over two sites with the major conformation having a site occupation factor of 0.720 (10). Similarity restraints with a tolerance of 0.01 Å were applied to the chemically equivalent bond lengths involving all disordered atoms, while neighbouring disordered atoms were restrained to have similar atomic displacement parameters. Seven low angle reflections were omitted on account of obscuration by the beam stop.

The amide and carboxylic acid H atoms were placed in the positions found in a difference Fourier map and were then refined isotropically. All other H atoms were placed in geometrically optimized positions and constrained to ride on their parent atoms with C—H = 0.95 (aromatic), 0.98 (methyl), 0.99 (methylene) or 1.00 (methine) Å and with *U*_iso_(H) = 1.5*U*_eq_(C) for the methyl groups and 1.2*U*_eq_(C) otherwise.

### Crystal data

**Table:**

C_28_H_38_N_4_O_8_·C_2_H_6_OS	*F*(000) = 1360
*M**_r_* = 636.76	*D*_x_ = 1.337 Mg m^−^^3^
Orthorhombic, *P*2_1_2_1_2_1_	Mo *K*α radiation, λ = 0.71073 Å
Hall symbol: P 2ac 2ab	Cell parameters from 5144 reflections
*a* = 10.8594 (1) Å	θ = 1.0–30.0°
*b* = 13.7414 (2) Å	µ = 0.16 mm^−^^1^
*c* = 21.1929 (3) Å	*T* = 160 K
*V* = 3162.48 (7) Å^3^	Prism, colourless
*Z* = 4	0.28 × 0.20 × 0.18 mm

### Data collection

**Table:**

Nonius KappaCCD area-detector diffractometer	7711 reflections with *I* > 2σ(*I*)
Radiation source: Nonius FR590 sealed tube generator	*R*_int_ = 0.044
Horizontally mounted graphite crystal monochromator	θ_max_ = 30.0°, θ_min_ = 2.1°
Detector resolution: 9 pixels mm^-1^	*h* = −15→15
φ and ω scans with κ offsets	*k* = −19→19
53400 measured reflections	*l* = −29→29
9238 independent reflections	

### Refinement

**Table:**

Refinement on *F*^2^	Hydrogen site location: difference Fourier map
Least-squares matrix: full	H atoms treated by a mixture of independent and constrained refinement
*R*[*F*^2^ > 2σ(*F*^2^)] = 0.042	*w* = 1/[σ^2^(*F*_o_^2^) + (0.0482*P*)^2^ + 0.4741*P*] where *P* = (*F*_o_^2^ + 2*F*_c_^2^)/3
*wR*(*F*^2^) = 0.103	(Δ/σ)_max_ = 0.003
*S* = 1.02	Δρ_max_ = 0.28 e Å^−^^3^
9231 reflections	Δρ_min_ = −0.33 e Å^−^^3^
433 parameters	Extinction correction: *SHELXL97* (Sheldrick, 2008), Fc^*^=kFc[1+0.001xFc^2^λ^3^/sin(2θ)]^-1/4^
21 restraints	Extinction coefficient: 0.0096 (11)
Primary atom site location: structure-invariant direct methods	Absolute structure: Flack & Bernardinelli (1999, 2000), 4115 Friedel pairs
Secondary atom site location: difference Fourier map	Flack parameter: −0.02 (8)

### Special details

**Table:**

Experimental. Solvent used: DMSO Cooling Device: Oxford Cryosystems Cryostream 700 Crystal mount: glued on a glass fibre Mosaicity (°.): 0.519 (1) Frames collected: 329 Seconds exposure per frame: 42 Degrees rotation per frame: 1.4 Crystal-Detector distance (mm): 30.0
Geometry. All e.s.d.'s (except the e.s.d. in the dihedral angle between two l.s. planes) are estimated using the full covariance matrix. The cell e.s.d.'s are taken into account individually in the estimation of e.s.d.'s in distances, angles and torsion angles; correlations between e.s.d.'s in cell parameters are only used when they are defined by crystal symmetry. An approximate (isotropic) treatment of cell e.s.d.'s is used for estimating e.s.d.'s involving l.s. planes.
Refinement. The structure contains one molecule of DMSO per peptide molecule. The *S* atom of the DMSO molecule is disordered over two sites with the major orientation having a site occupation factor of 0.879 (2). One C atom of the central five-membered ring of the peptide molecule is also disordered over two sites with the major conformation having a site occupation factor of 0.720 (10). Similarity restraints with a tolerance of 0.01 Å were applied to the chemically equivalent bond lengths involving all disordered atoms, while neighbouring disordered atoms were restrained to have similar atomic displacement parameters. Seven low angle reflections were omitted on account of obscuration by the beam stop.

### Fractional atomic coordinates and isotropic or equivalent isotropic displacement parameters (Å^2^)

**Table:**

	*x*	*y*	*z*	*U*_iso_*/*U*_eq_	Occ. (<1)
O1	0.49592 (11)	0.79228 (10)	0.02238 (7)	0.0429 (3)	
O2	0.38389 (11)	0.88646 (9)	−0.04216 (5)	0.0320 (3)	
H2	0.456 (2)	0.9100 (18)	−0.0512 (11)	0.050 (6)*	
O4	0.30778 (11)	0.60218 (9)	−0.01081 (5)	0.0320 (3)	
O7	0.08885 (11)	0.52449 (9)	0.08052 (5)	0.0329 (3)	
O10	0.17612 (11)	0.31930 (8)	0.20810 (5)	0.0316 (3)	
O13	0.39163 (11)	0.45955 (9)	0.26319 (5)	0.0324 (3)	
O19	0.57060 (13)	0.44020 (11)	0.04583 (8)	0.0516 (4)	
O30	0.71136 (12)	0.69569 (9)	0.46113 (5)	0.0366 (3)	
N3	0.28614 (13)	0.70479 (10)	0.07067 (6)	0.0276 (3)	
N6	0.25499 (12)	0.52124 (9)	0.14416 (6)	0.0249 (3)	
H6	0.2849 (19)	0.5049 (15)	0.1807 (10)	0.039 (5)*	
N9	0.11523 (12)	0.46304 (9)	0.24731 (6)	0.0258 (3)	
N12	0.28842 (13)	0.41680 (10)	0.35078 (7)	0.0293 (3)	
H12	0.286 (2)	0.4175 (16)	0.3887 (11)	0.043 (6)*	
C1	0.39833 (15)	0.81984 (11)	0.00303 (7)	0.0276 (3)	
C2	0.27251 (15)	0.78716 (11)	0.02743 (7)	0.0275 (3)	
H1	0.2165	0.7701	−0.0084	0.033*	
C4	0.30659 (14)	0.61553 (11)	0.04668 (7)	0.0260 (3)	
C5	0.34313 (14)	0.53169 (12)	0.09183 (7)	0.0260 (3)	
C7	0.13440 (14)	0.51093 (11)	0.13341 (7)	0.0246 (3)	
C8	0.05129 (14)	0.48351 (13)	0.18813 (7)	0.0289 (3)	
H8	0.0017	0.4251	0.1760	0.035*	
C10	0.17081 (14)	0.37502 (11)	0.25323 (7)	0.0254 (3)	
C11	0.21698 (15)	0.34116 (11)	0.31820 (7)	0.0277 (3)	
C13	0.37882 (14)	0.46585 (12)	0.32135 (7)	0.0271 (3)	
C14	0.21553 (17)	0.86652 (13)	0.06872 (8)	0.0364 (4)	
H141	0.2390	0.9322	0.0537	0.044*	
H142	0.1246	0.8613	0.0695	0.044*	
C15	0.2703 (2)	0.84566 (13)	0.13333 (9)	0.0399 (4)	
H151	0.2200	0.8752	0.1673	0.048*	
H152	0.3557	0.8704	0.1364	0.048*	
C16	0.26708 (18)	0.73510 (12)	0.13691 (7)	0.0339 (4)	
H161	0.3335	0.7101	0.1645	0.041*	
H162	0.1868	0.7118	0.1530	0.041*	
C17	0.47177 (16)	0.55084 (14)	0.11973 (8)	0.0335 (4)	
H171	0.4754	0.6182	0.1362	0.040*	
H172	0.4863	0.5058	0.1554	0.040*	
C18	0.57147 (17)	0.53701 (16)	0.07076 (10)	0.0443 (5)	
H181	0.5590	0.5841	0.0360	0.053*	
H182	0.6527	0.5504	0.0901	0.053*	
C20	0.45803 (19)	0.42227 (16)	0.01315 (10)	0.0456 (5)	
H201	0.4592	0.3554	−0.0042	0.055*	
H202	0.4507	0.4682	−0.0226	0.055*	
C21	0.34751 (16)	0.43379 (12)	0.05612 (8)	0.0329 (4)	
H211	0.3479	0.3802	0.0873	0.039*	
H212	0.2717	0.4273	0.0305	0.039*	
C22	−0.0366 (2)	0.56707 (17)	0.20421 (10)	0.0494 (5)	
H221	−0.0074	0.6287	0.1851	0.059*	0.720 (10)
H222	−0.1204	0.5531	0.1882	0.059*	0.720 (10)
H223	−0.0384	0.6157	0.1698	0.059*	0.280 (10)
H224	−0.1212	0.5424	0.2111	0.059*	0.280 (10)
C23	−0.0374 (3)	0.5746 (3)	0.27560 (17)	0.0433 (9)	0.720 (10)
H231	−0.0500	0.6428	0.2893	0.052*	0.720 (10)
H232	−0.1031	0.5335	0.2940	0.052*	0.720 (10)
C23A	0.0100 (10)	0.6075 (6)	0.2588 (4)	0.040 (2)	0.280 (10)
H233	−0.0592	0.6288	0.2860	0.048*	0.280 (10)
H234	0.0591	0.6658	0.2479	0.048*	0.280 (10)
C24	0.08878 (18)	0.53834 (13)	0.29483 (9)	0.0380 (4)	
H241	0.1505	0.5913	0.2932	0.046*	0.720 (10)
H242	0.0873	0.5104	0.3379	0.046*	0.720 (10)
H243	0.1653	0.5701	0.3097	0.046*	0.280 (10)
H244	0.0441	0.5109	0.3315	0.046*	0.280 (10)
C25	0.29830 (18)	0.25073 (13)	0.31024 (9)	0.0385 (4)	
H251	0.3699	0.2671	0.2842	0.058*	
H252	0.2508	0.1990	0.2897	0.058*	
H253	0.3261	0.2283	0.3518	0.058*	
C26	0.10223 (17)	0.31609 (13)	0.35741 (8)	0.0345 (4)	
H261	0.1270	0.2801	0.3953	0.052*	
H262	0.0463	0.2759	0.3322	0.052*	
H263	0.0602	0.3762	0.3698	0.052*	
C27	0.46259 (14)	0.52808 (12)	0.35961 (7)	0.0264 (3)	
C28	0.45392 (15)	0.53814 (13)	0.42525 (8)	0.0298 (3)	
H28	0.3900	0.5061	0.4477	0.036*	
C29	0.53853 (16)	0.59476 (13)	0.45724 (8)	0.0321 (4)	
H29	0.5324	0.6014	0.5017	0.039*	
C30	0.63240 (15)	0.64214 (12)	0.42502 (8)	0.0294 (3)	
C31	0.64158 (15)	0.63358 (13)	0.35972 (8)	0.0315 (4)	
H31	0.7049	0.6664	0.3373	0.038*	
C32	0.55693 (15)	0.57640 (13)	0.32804 (8)	0.0305 (3)	
H32	0.5633	0.5699	0.2835	0.037*	
C33	0.82609 (16)	0.72279 (15)	0.43330 (9)	0.0380 (4)	
H331	0.8678	0.6646	0.4173	0.057*	
H332	0.8780	0.7544	0.4651	0.057*	
H333	0.8112	0.7681	0.3984	0.057*	
S1A	0.73887 (6)	0.66072 (5)	0.67871 (3)	0.0570 (2)	0.879 (2)
S1B	0.7574 (3)	0.7383 (4)	0.69153 (18)	0.0500 (16)	0.121 (2)
O3	0.66114 (17)	0.7045 (2)	0.72672 (8)	0.0883 (7)	
C34	0.7137 (4)	0.7294 (3)	0.60964 (12)	0.1115 (14)	
H341	0.6289	0.7197	0.5951	0.167*	0.879 (2)
H342	0.7711	0.7082	0.5767	0.167*	0.879 (2)
H343	0.7271	0.7985	0.6187	0.167*	0.879 (2)
H344	0.6299	0.7546	0.6042	0.167*	0.121 (2)
H345	0.7166	0.6612	0.5963	0.167*	0.121 (2)
H346	0.7709	0.7677	0.5839	0.167*	0.121 (2)
C35	0.8931 (2)	0.6951 (2)	0.69070 (15)	0.0747 (8)	
H351	0.9250	0.6630	0.7286	0.112*	0.879 (2)
H352	0.8979	0.7658	0.6960	0.112*	0.879 (2)
H353	0.9425	0.6756	0.6541	0.112*	0.879 (2)
H354	0.9269	0.6957	0.7336	0.112*	0.121 (2)
H355	0.9452	0.7349	0.6631	0.112*	0.121 (2)
H356	0.8911	0.6281	0.6749	0.112*	0.121 (2)

### Atomic displacement parameters (Å^2^)

**Table:**

	*U*^11^	*U*^22^	*U*^33^	*U*^12^	*U*^13^	*U*^23^
O1	0.0304 (6)	0.0456 (8)	0.0528 (8)	0.0006 (6)	0.0038 (6)	0.0157 (6)
O2	0.0334 (6)	0.0334 (6)	0.0292 (6)	−0.0044 (5)	0.0053 (5)	0.0061 (5)
O4	0.0417 (7)	0.0340 (6)	0.0204 (5)	−0.0006 (5)	0.0028 (5)	−0.0003 (4)
O7	0.0323 (6)	0.0417 (7)	0.0249 (6)	0.0017 (5)	−0.0070 (5)	0.0045 (5)
O10	0.0406 (7)	0.0295 (6)	0.0247 (5)	0.0027 (5)	0.0021 (5)	−0.0036 (5)
O13	0.0326 (6)	0.0434 (7)	0.0213 (5)	−0.0044 (5)	0.0012 (5)	0.0016 (5)
O19	0.0385 (7)	0.0555 (9)	0.0608 (9)	0.0109 (6)	0.0117 (6)	−0.0010 (7)
O30	0.0419 (7)	0.0399 (7)	0.0281 (6)	−0.0096 (6)	−0.0046 (5)	0.0007 (5)
N3	0.0356 (7)	0.0259 (7)	0.0214 (6)	−0.0028 (5)	0.0028 (5)	0.0005 (5)
N6	0.0276 (6)	0.0275 (6)	0.0195 (6)	−0.0026 (5)	−0.0014 (5)	0.0036 (5)
N9	0.0310 (6)	0.0250 (6)	0.0214 (6)	0.0023 (5)	0.0025 (5)	0.0016 (5)
N12	0.0345 (7)	0.0341 (7)	0.0194 (6)	−0.0048 (6)	−0.0010 (6)	0.0029 (6)
C1	0.0322 (8)	0.0264 (8)	0.0242 (7)	−0.0015 (6)	0.0056 (6)	−0.0010 (6)
C2	0.0307 (8)	0.0253 (7)	0.0266 (8)	−0.0024 (6)	0.0031 (6)	0.0031 (6)
C4	0.0279 (8)	0.0279 (8)	0.0221 (7)	−0.0032 (6)	0.0024 (6)	0.0000 (6)
C5	0.0281 (8)	0.0280 (8)	0.0218 (7)	−0.0029 (6)	0.0021 (6)	0.0005 (6)
C7	0.0276 (8)	0.0231 (7)	0.0232 (7)	0.0001 (6)	−0.0037 (6)	0.0015 (6)
C8	0.0248 (7)	0.0344 (9)	0.0274 (8)	0.0021 (6)	−0.0012 (6)	0.0050 (7)
C10	0.0276 (7)	0.0266 (8)	0.0221 (7)	−0.0026 (6)	0.0041 (6)	0.0021 (6)
C11	0.0344 (8)	0.0260 (7)	0.0229 (7)	−0.0037 (6)	−0.0001 (6)	0.0037 (6)
C13	0.0281 (7)	0.0302 (8)	0.0231 (7)	0.0015 (6)	0.0001 (6)	0.0033 (6)
C14	0.0397 (9)	0.0306 (9)	0.0389 (9)	0.0019 (7)	0.0131 (8)	0.0026 (7)
C15	0.0564 (12)	0.0291 (8)	0.0341 (9)	−0.0044 (8)	0.0100 (8)	−0.0038 (7)
C16	0.0487 (10)	0.0314 (8)	0.0215 (7)	−0.0036 (7)	0.0060 (7)	−0.0034 (6)
C17	0.0288 (8)	0.0390 (9)	0.0328 (9)	−0.0037 (7)	−0.0002 (7)	0.0025 (7)
C18	0.0309 (9)	0.0523 (12)	0.0498 (11)	−0.0022 (8)	0.0063 (8)	0.0066 (9)
C20	0.0495 (11)	0.0428 (11)	0.0444 (11)	0.0071 (9)	0.0105 (9)	−0.0059 (9)
C21	0.0377 (9)	0.0275 (8)	0.0334 (9)	0.0018 (7)	−0.0003 (7)	−0.0023 (7)
C22	0.0458 (11)	0.0591 (13)	0.0433 (11)	0.0242 (10)	0.0071 (9)	0.0088 (10)
C23	0.0353 (17)	0.0476 (19)	0.0471 (18)	0.0101 (14)	0.0154 (13)	0.0004 (14)
C23A	0.034 (4)	0.043 (4)	0.042 (4)	0.018 (3)	0.011 (3)	−0.007 (3)
C24	0.0481 (10)	0.0303 (9)	0.0355 (9)	0.0071 (8)	0.0044 (8)	−0.0065 (7)
C25	0.0419 (9)	0.0324 (9)	0.0412 (10)	0.0041 (7)	−0.0051 (8)	0.0058 (8)
C26	0.0391 (9)	0.0387 (9)	0.0255 (8)	−0.0110 (7)	0.0016 (7)	0.0050 (7)
C27	0.0286 (7)	0.0283 (8)	0.0224 (7)	0.0036 (6)	−0.0003 (6)	0.0031 (6)
C28	0.0317 (8)	0.0329 (8)	0.0249 (7)	−0.0012 (7)	0.0028 (6)	0.0025 (7)
C29	0.0390 (9)	0.0351 (9)	0.0221 (8)	−0.0036 (7)	0.0025 (7)	−0.0005 (7)
C30	0.0333 (8)	0.0287 (8)	0.0262 (8)	−0.0011 (6)	−0.0048 (6)	0.0011 (6)
C31	0.0318 (8)	0.0359 (9)	0.0269 (8)	−0.0043 (7)	0.0000 (6)	0.0058 (7)
C32	0.0331 (8)	0.0363 (9)	0.0222 (7)	−0.0020 (7)	−0.0006 (6)	0.0028 (7)
C33	0.0332 (9)	0.0434 (10)	0.0372 (10)	−0.0028 (7)	−0.0073 (7)	0.0011 (8)
S1A	0.0586 (4)	0.0605 (5)	0.0519 (4)	−0.0202 (3)	−0.0122 (3)	0.0035 (3)
S1B	0.043 (2)	0.068 (3)	0.039 (2)	−0.002 (2)	−0.0026 (17)	−0.017 (2)
O3	0.0570 (10)	0.161 (2)	0.0465 (10)	−0.0138 (13)	0.0028 (8)	0.0158 (12)
C34	0.151 (4)	0.152 (3)	0.0315 (12)	0.050 (3)	−0.0083 (17)	0.0009 (17)
C35	0.0509 (13)	0.0781 (18)	0.095 (2)	−0.0069 (13)	0.0034 (14)	−0.0142 (16)

### Geometric parameters (Å, º)

**Table:**

O1—C1	1.198 (2)	C22—C23A	1.381 (8)
O2—C1	1.334 (2)	C22—C23	1.517 (4)
O2—H2	0.87 (2)	C22—H221	0.9900
O4—C4	1.2320 (19)	C22—H222	0.9900
O7—C7	1.2393 (18)	C22—H223	0.9900
O10—C10	1.2265 (19)	C22—H224	0.9900
O13—C13	1.2434 (19)	C23—C24	1.514 (3)
O19—C20	1.426 (3)	C23—H231	0.9900
O19—C18	1.431 (3)	C23—H232	0.9900
O30—C30	1.365 (2)	C23A—C24	1.489 (6)
O30—C33	1.428 (2)	C23A—H233	0.9900
N3—C4	1.346 (2)	C23A—H234	0.9900
N3—C2	1.464 (2)	C24—H241	0.9900
N3—C16	1.479 (2)	C24—H242	0.9900
N6—C7	1.337 (2)	C24—H243	0.9900
N6—C5	1.4721 (19)	C24—H244	0.9900
N6—H6	0.87 (2)	C25—H251	0.9800
N9—C10	1.357 (2)	C25—H252	0.9800
N9—C8	1.461 (2)	C25—H253	0.9800
N9—C24	1.472 (2)	C26—H261	0.9800
N12—C13	1.344 (2)	C26—H262	0.9800
N12—C11	1.469 (2)	C26—H263	0.9800
N12—H12	0.80 (2)	C27—C32	1.392 (2)
C1—C2	1.528 (2)	C27—C28	1.401 (2)
C2—C14	1.529 (2)	C28—C29	1.382 (2)
C2—H1	1.0000	C28—H28	0.9500
C4—C5	1.549 (2)	C29—C30	1.389 (2)
C5—C17	1.540 (2)	C29—H29	0.9500
C5—C21	1.544 (2)	C30—C31	1.393 (2)
C7—C8	1.517 (2)	C31—C32	1.383 (2)
C8—C22	1.531 (3)	C31—H31	0.9500
C8—H8	1.0000	C32—H32	0.9500
C10—C11	1.537 (2)	C33—H331	0.9800
C11—C25	1.534 (2)	C33—H332	0.9800
C11—C26	1.537 (2)	C33—H333	0.9800
C13—C27	1.489 (2)	S1A—O3	1.452 (2)
C14—C15	1.520 (3)	S1A—C35	1.759 (3)
C14—H141	0.9900	S1A—C34	1.763 (3)
C14—H142	0.9900	S1B—O3	1.366 (4)
C15—C16	1.522 (2)	S1B—C35	1.589 (4)
C15—H151	0.9900	S1B—C34	1.803 (4)
C15—H152	0.9900	C34—H341	0.9800
C16—H161	0.9900	C34—H342	0.9800
C16—H162	0.9900	C34—H343	0.9800
C17—C18	1.512 (3)	C34—H344	0.9800
C17—H171	0.9900	C34—H345	0.9800
C17—H172	0.9900	C34—H346	0.9800
C18—H181	0.9900	C35—H351	0.9800
C18—H182	0.9900	C35—H352	0.9800
C20—C21	1.515 (3)	C35—H353	0.9800
C20—H201	0.9900	C35—H354	0.9800
C20—H202	0.9900	C35—H355	0.9800
C21—H211	0.9900	C35—H356	0.9800
C21—H212	0.9900		
			
C1—O2—H2	108.0 (16)	C8—C22—H221	110.5
C20—O19—C18	110.20 (15)	C23—C22—H222	110.5
C30—O30—C33	117.22 (13)	C8—C22—H222	110.5
C4—N3—C2	118.99 (12)	H221—C22—H222	108.7
C4—N3—C16	129.66 (13)	C23A—C22—H223	110.7
C2—N3—C16	111.25 (13)	C8—C22—H223	110.7
C7—N6—C5	121.26 (13)	C23A—C22—H224	110.7
C7—N6—H6	119.4 (13)	C8—C22—H224	110.7
C5—N6—H6	117.0 (13)	H223—C22—H224	108.8
C10—N9—C8	117.53 (13)	C24—C23—C22	103.9 (2)
C10—N9—C24	130.52 (13)	C24—C23—H231	111.0
C8—N9—C24	111.05 (13)	C22—C23—H231	111.0
C13—N12—C11	121.47 (14)	C24—C23—H232	111.0
C13—N12—H12	119.0 (16)	C22—C23—H232	111.0
C11—N12—H12	117.3 (16)	H231—C23—H232	109.0
O1—C1—O2	124.53 (15)	C22—C23A—C24	112.5 (5)
O1—C1—C2	125.60 (14)	C22—C23A—H233	109.1
O2—C1—C2	109.84 (14)	C24—C23A—H233	109.1
N3—C2—C1	110.40 (13)	C22—C23A—H234	109.1
N3—C2—C14	103.54 (12)	C24—C23A—H234	109.1
C1—C2—C14	110.24 (13)	H233—C23A—H234	107.8
N3—C2—H1	110.8	N9—C24—C23A	102.1 (3)
C1—C2—H1	110.8	N9—C24—C23	102.94 (17)
C14—C2—H1	110.8	N9—C24—H241	111.2
O4—C4—N3	120.73 (14)	C23—C24—H241	111.2
O4—C4—C5	119.81 (14)	N9—C24—H242	111.2
N3—C4—C5	119.11 (13)	C23—C24—H242	111.2
N6—C5—C17	108.49 (12)	H241—C24—H242	109.1
N6—C5—C21	107.72 (13)	N9—C24—H243	111.3
C17—C5—C21	108.02 (13)	C23A—C24—H243	111.3
N6—C5—C4	111.80 (13)	N9—C24—H244	111.3
C17—C5—C4	110.03 (13)	C23A—C24—H244	111.3
C21—C5—C4	110.67 (13)	H243—C24—H244	109.2
O7—C7—N6	121.95 (15)	C11—C25—H251	109.5
O7—C7—C8	119.43 (14)	C11—C25—H252	109.5
N6—C7—C8	118.61 (13)	H251—C25—H252	109.5
N9—C8—C7	114.92 (12)	C11—C25—H253	109.5
N9—C8—C22	104.45 (14)	H251—C25—H253	109.5
C7—C8—C22	110.77 (14)	H252—C25—H253	109.5
N9—C8—H8	108.8	C11—C26—H261	109.5
C7—C8—H8	108.8	C11—C26—H262	109.5
C22—C8—H8	108.8	H261—C26—H262	109.5
O10—C10—N9	120.34 (14)	C11—C26—H263	109.5
O10—C10—C11	119.60 (14)	H261—C26—H263	109.5
N9—C10—C11	119.83 (13)	H262—C26—H263	109.5
N12—C11—C25	108.71 (14)	C32—C27—C28	118.65 (15)
N12—C11—C26	109.42 (13)	C32—C27—C13	117.50 (13)
C25—C11—C26	110.16 (14)	C28—C27—C13	123.82 (14)
N12—C11—C10	112.28 (12)	C29—C28—C27	119.87 (15)
C25—C11—C10	109.54 (13)	C29—C28—H28	120.1
C26—C11—C10	106.72 (13)	C27—C28—H28	120.1
O13—C13—N12	120.45 (15)	C28—C29—C30	120.72 (15)
O13—C13—C27	120.77 (14)	C28—C29—H29	119.6
N12—C13—C27	118.77 (14)	C30—C29—H29	119.6
C15—C14—C2	102.88 (14)	O30—C30—C29	116.00 (14)
C15—C14—H141	111.2	O30—C30—C31	123.90 (15)
C2—C14—H141	111.2	C29—C30—C31	120.10 (15)
C15—C14—H142	111.2	C32—C31—C30	118.86 (15)
C2—C14—H142	111.2	C32—C31—H31	120.6
H141—C14—H142	109.1	C30—C31—H31	120.6
C14—C15—C16	102.97 (15)	C31—C32—C27	121.79 (15)
C14—C15—H151	111.2	C31—C32—H32	119.1
C16—C15—H151	111.2	C27—C32—H32	119.1
C14—C15—H152	111.2	O30—C33—H331	109.5
C16—C15—H152	111.2	O30—C33—H332	109.5
H151—C15—H152	109.1	H331—C33—H332	109.5
N3—C16—C15	103.33 (13)	O30—C33—H333	109.5
N3—C16—H161	111.1	H331—C33—H333	109.5
C15—C16—H161	111.1	H332—C33—H333	109.5
N3—C16—H162	111.1	O3—S1A—C35	109.95 (13)
C15—C16—H162	111.1	O3—S1A—C34	105.67 (16)
H161—C16—H162	109.1	C35—S1A—C34	97.1 (2)
C18—C17—C5	111.38 (14)	O3—S1B—C35	126.1 (4)
C18—C17—H171	109.4	O3—S1B—C34	107.5 (3)
C5—C17—H171	109.4	C35—S1B—C34	102.0 (3)
C18—C17—H172	109.4	S1A—C34—H341	109.5
C5—C17—H172	109.4	S1A—C34—H342	109.5
H171—C17—H172	108.0	H341—C34—H342	109.5
O19—C18—C17	111.44 (16)	S1A—C34—H343	109.5
O19—C18—H181	109.3	H341—C34—H343	109.5
C17—C18—H181	109.3	H342—C34—H343	109.5
O19—C18—H182	109.3	S1B—C34—H344	109.5
C17—C18—H182	109.3	S1B—C34—H345	109.5
H181—C18—H182	108.0	H344—C34—H345	109.5
O19—C20—C21	111.66 (15)	S1B—C34—H346	109.5
O19—C20—H201	109.3	H344—C34—H346	109.5
C21—C20—H201	109.3	H345—C34—H346	109.5
O19—C20—H202	109.3	S1A—C35—H351	109.5
C21—C20—H202	109.3	S1A—C35—H352	109.5
H201—C20—H202	107.9	H351—C35—H352	109.5
C20—C21—C5	114.20 (15)	S1A—C35—H353	109.5
C20—C21—H211	108.7	H351—C35—H353	109.5
C5—C21—H211	108.7	H352—C35—H353	109.5
C20—C21—H212	108.7	S1B—C35—H354	109.5
C5—C21—H212	108.7	S1B—C35—H355	109.5
H211—C21—H212	107.6	H354—C35—H355	109.5
C23A—C22—C8	105.1 (3)	S1B—C35—H356	109.5
C23—C22—C8	106.09 (17)	H354—C35—H356	109.5
C23—C22—H221	110.5	H355—C35—H356	109.5
			
C4—N3—C2—C1	−77.56 (18)	C21—C5—C17—C18	49.02 (19)
C16—N3—C2—C1	105.76 (15)	C4—C5—C17—C18	−71.89 (18)
C4—N3—C2—C14	164.46 (14)	C20—O19—C18—C17	63.9 (2)
C16—N3—C2—C14	−12.22 (18)	C5—C17—C18—O19	−59.5 (2)
O1—C1—C2—N3	−9.7 (2)	C18—O19—C20—C21	−59.6 (2)
O2—C1—C2—N3	172.24 (12)	O19—C20—C21—C5	52.7 (2)
O1—C1—C2—C14	104.13 (19)	N6—C5—C21—C20	−163.52 (15)
O2—C1—C2—C14	−73.97 (17)	C17—C5—C21—C20	−46.52 (19)
C2—N3—C4—O4	−3.6 (2)	C4—C5—C21—C20	73.99 (18)
C16—N3—C4—O4	172.40 (16)	N9—C8—C22—C23A	19.5 (6)
C2—N3—C4—C5	169.63 (13)	C7—C8—C22—C23A	−104.8 (6)
C16—N3—C4—C5	−14.4 (2)	N9—C8—C22—C23	−12.2 (3)
C7—N6—C5—C17	175.20 (14)	C7—C8—C22—C23	−136.4 (2)
C7—N6—C5—C21	−68.11 (18)	C23A—C22—C23—C24	−63.7 (4)
C7—N6—C5—C4	53.69 (19)	C8—C22—C23—C24	28.7 (3)
O4—C4—C5—N6	−133.33 (15)	C23—C22—C23A—C24	73.4 (7)
N3—C4—C5—N6	53.40 (19)	C8—C22—C23A—C24	−22.8 (9)
O4—C4—C5—C17	106.06 (17)	C10—N9—C24—C23A	−171.4 (5)
N3—C4—C5—C17	−67.22 (18)	C8—N9—C24—C23A	−2.8 (5)
O4—C4—C5—C21	−13.2 (2)	C10—N9—C24—C23	−140.9 (2)
N3—C4—C5—C21	173.48 (14)	C8—N9—C24—C23	27.7 (3)
C5—N6—C7—O7	−11.3 (2)	C22—C23A—C24—N9	16.5 (9)
C5—N6—C7—C8	169.78 (14)	C22—C23A—C24—C23	−78.4 (10)
C10—N9—C8—C7	−78.00 (18)	C22—C23—C24—N9	−33.8 (3)
C24—N9—C8—C7	111.71 (16)	C22—C23—C24—C23A	58.0 (6)
C10—N9—C8—C22	160.44 (15)	O13—C13—C27—C32	−1.6 (2)
C24—N9—C8—C22	−9.85 (19)	N12—C13—C27—C32	178.69 (15)
O7—C7—C8—N9	176.18 (14)	O13—C13—C27—C28	−179.64 (16)
N6—C7—C8—N9	−4.9 (2)	N12—C13—C27—C28	0.7 (2)
O7—C7—C8—C22	−65.8 (2)	C32—C27—C28—C29	−0.3 (2)
N6—C7—C8—C22	113.20 (17)	C13—C27—C28—C29	177.63 (16)
C8—N9—C10—O10	6.6 (2)	C27—C28—C29—C30	0.1 (3)
C24—N9—C10—O10	174.63 (16)	C33—O30—C30—C29	163.21 (15)
C8—N9—C10—C11	−167.91 (13)	C33—O30—C30—C31	−16.9 (2)
C24—N9—C10—C11	0.1 (2)	C28—C29—C30—O30	−179.63 (16)
C13—N12—C11—C25	73.25 (18)	C28—C29—C30—C31	0.5 (3)
C13—N12—C11—C26	−166.40 (15)	O30—C30—C31—C32	179.34 (16)
C13—N12—C11—C10	−48.1 (2)	C29—C30—C31—C32	−0.8 (3)
O10—C10—C11—N12	137.80 (15)	C30—C31—C32—C27	0.5 (3)
N9—C10—C11—N12	−47.67 (19)	C28—C27—C32—C31	0.0 (2)
O10—C10—C11—C25	16.9 (2)	C13—C27—C32—C31	−178.07 (15)
N9—C10—C11—C25	−168.55 (14)	C35—S1B—O3—S1A	−59.6 (3)
O10—C10—C11—C26	−102.32 (17)	C34—S1B—O3—S1A	60.4 (2)
N9—C10—C11—C26	72.21 (17)	C35—S1A—O3—S1B	42.1 (3)
C11—N12—C13—O13	11.4 (2)	C34—S1A—O3—S1B	−61.7 (3)
C11—N12—C13—C27	−168.90 (14)	O3—S1A—C34—S1B	54.6 (2)
N3—C2—C14—C15	32.26 (17)	C35—S1A—C34—S1B	−58.5 (2)
C1—C2—C14—C15	−85.84 (16)	O3—S1B—C34—S1A	−61.0 (3)
C2—C14—C15—C16	−40.31 (18)	C35—S1B—C34—S1A	73.3 (3)
C4—N3—C16—C15	171.04 (17)	O3—S1B—C35—S1A	56.1 (3)
C2—N3—C16—C15	−12.74 (19)	C34—S1B—C35—S1A	−66.3 (2)
C14—C15—C16—N3	32.57 (19)	O3—S1A—C35—S1B	−42.1 (3)
N6—C5—C17—C18	165.52 (15)	C34—S1A—C35—S1B	67.4 (3)

### Hydrogen-bond geometry (Å, º)

**Table:**

*D*—H···*A*	*D*—H	H···*A*	*D*···*A*	*D*—H···*A*
O2—H2···O7^i^	0.87 (2)	1.81 (3)	2.6669 (17)	168 (2)
N6—H6···O13	0.87 (2)	2.19 (2)	3.0468 (17)	169.5 (18)
N12—H12···O4^ii^	0.80 (2)	2.37 (2)	3.1247 (18)	156 (2)

Symmetry codes: (i) *x*+1/2, −*y*+3/2, −*z*; (ii) −*x*+1/2, −*y*+1, *z*+1/2.
